# Malaria resurgence: a systematic review and assessment of its causes

**DOI:** 10.1186/1475-2875-11-122

**Published:** 2012-04-24

**Authors:** Justin M Cohen, David L Smith, Chris Cotter, Abigail Ward, Gavin Yamey, Oliver J Sabot, Bruno Moonen

**Affiliations:** 1 Clinton Health Access Initiative, Boston, MA, USA; 2 Johns Hopkins Malaria Research Institute and Department of Epidemiology, Baltimore, MD, USA; 3 The Center for Disease Dynamics, Economics & Policy, Washington, DC, USA; 4 The Global Health Group, University of California, San Francisco, San Francisco, CA, USA

## Abstract

**Background:**

Considerable declines in malaria have accompanied increased funding for control since the year 2000, but historical failures to maintain gains against the disease underscore the fragility of these successes. Although malaria transmission can be suppressed by effective control measures, in the absence of active intervention malaria will return to an intrinsic equilibrium determined by factors related to ecology, efficiency of mosquito vectors, and socioeconomic characteristics. Understanding where and why resurgence has occurred historically can help current and future malaria control programmes avoid the mistakes of the past.

**Methods:**

A systematic review of the literature was conducted to identify historical malaria resurgence events. All suggested causes of these events were categorized according to whether they were related to weakened malaria control programmes, increased potential for malaria transmission, or technical obstacles like resistance.

**Results:**

The review identified 75 resurgence events in 61 countries, occurring from the 1930s through the 2000s. Almost all resurgence events (68/75 = 91%) were attributed at least in part to the weakening of malaria control programmes for a variety of reasons, of which resource constraints were the most common (39/68 = 57%). Over half of the events (44/75 = 59%) were attributed in part to increases in the intrinsic potential for malaria transmission, while only 24/75 (32%) were attributed to vector or drug resistance.

**Conclusions:**

Given that most malaria resurgences have been linked to weakening of control programmes, there is an urgent need to develop practical solutions to the financial and operational threats to effectively sustaining today’s successful malaria control programmes.

## Background

The gains achieved against malaria in the past decade have no parallel since the Global Malaria Eradication Programme (GMEP), which ended in 1969 [1]. Increased funding since 2000 has allowed scale-up of effective interventions, and malaria has declined considerably in many previously highly endemic parts of the world [2]. While these successes confirm that well-funded anti-malaria interventions can have enormous impact, the global increase in malaria burden that occurred in the aftermath of the GMEP [3] underscores the potential fragility of such gains. In 1972, when malaria was on the rise after cessation of the GMEP, Bruce-Chwatt suggested the term “resurgence” to refer to “the reappearance of new infections in significant numbers after malaria has subsided owing to the measures applied to reduce or interrupt its transmission” [4]. Nájera later clarified, “A malaria resurgence is actually the return to a state of equilibrium which has been disturbed” [5] by malaria control efforts.

Resurgence is the result of the fact that there is a certain intrinsic potential for malaria in an area, mathematically described by the basic reproduction number R_0_[6]. Although malaria can be reduced from that baseline by implementation of effective control measures, in the absence of active suppression malaria will return to a prevalence level determined by R_0_. This intrinsic potential for malaria transmission may evolve slowly as a function of socioeconomic development or environmental change. Such structural changes may eventually result in sufficiently low potential that active measures are not required to suppress transmission, but the malaria baseline will usually be unaffected by commonly implemented malaria control activities [7]. The concept of resurgence as a return towards a baseline level of malaria is distinct from that of “rebound” [8], which is used to describe a hypothetical overshoot that could occur in populations that have lost their immunity.

Today, the threat of resurgence again looms as constrained global funding and competing priorities threaten the sustainability of successes [9,10]. Brief increases in malaria incidence in countries including Rwanda and Zambia have raised fears about whether recent gains against malaria can be sustained and extended [2]. At the same time, it has been suggested that technical problems—such as insecticide resistance and reduced effectiveness of insecticide-treated nets—may complicate continued progress in countries including Kenya [11] and Senegal [12]. Ensuring that today’s successful malaria programmes learn from history rather than repeat its mistakes requires a careful accounting of what has gone wrong in the past and an understanding of the factors that have driven those failures, whether technical, operational, or financial. Accordingly, a systematic literature review was conducted to identify all documented malaria resurgence events and the causes to which they have been attributed.

## Methods

### **Search strategy and selection criteria**

The electronic databases PubMed, Web of Knowledge, Scopus, and the World Health Organization’s WHOLIS and regional office databases were searched for articles documenting historical malaria resurgence events using the search terms “malaria” and either “resurg*”, “reemerg*”, or “re-emerg*” (wild-card operators were used to ensure that the search would identify “resurging”, “resurgence”, and any other form of the word). The searches, conducted on Aug 1, 2011, included references published on any date up until the day of the search and included those published in English, French, or Spanish. All records resulting from these searches were screened, and full-text articles were assessed if the reference appeared to describe or allude to a resurgence event. In addition, the reference lists of all articles for which the full text was reviewed were hand-searched, and the full text of those references that appeared relevant to malaria resurgence were retrieved.

Full-text articles were read to evaluate whether they included mention of any resurgence event. Although the term “resurgence” is sometimes used in a general, non-specific way to refer to any increase in malaria, this review defined a resurgence event more narrowly as:

An increasing trend in malaria incidence or prevalence following suppression achieved through implementation of control efforts.

Accordingly, any report of an increase in malaria incidence or prevalence in assessed articles was included in analysis if it appeared to a) involve an increase over a period of more than a single year or transmission season (i.e., there was a upward “trend” over time and not just a single aberrant season), and b) occur in a region where endemic malaria had previously been reported but where transmission had subsequently been suppressed to some degree through anti-malarial interventions. Any reference to such an event, whether national or subnational, was recorded, regardless of article type or quality.

### **Evaluation of causes**

After compiling all resurgence events in the identified citations that met these criteria, the same articles were reviewed for suggested causes of those resurgence events. Additionally, to identify potential causes suggested elsewhere, the same databases as above were searched for the name of the country involved and “malaria.” Search results were limited to articles published within a few years after the start of the resurgence event. The full-text of all results of these searches that appeared likely to discuss potential causes of resurgence was read, and the reference lists of these articles were hand-searched for relevant sources.

A data extraction form was used by two reviewers to classify all suggested causes for resurgence into categories. Three overarching categories were used to classify causes: 1) weakening of the malaria programme, 2) increasing intrinsic potential for malaria transmission, and 3) technical problems such as insecticide or drug resistance. All suggested causes for resurgence were recorded from each article, regardless of article type or quality of evidence. Additionally, however, each suggested cause was classified in regard to the degree of supporting evidence into one of two levels: a) assertions, without quantitative analysis or detailed argument for why that factor was a cause of resurgence, or b) evidence-based claims, where in-depth qualitative or quantitative analysis was used to provide evidence in support of the factor as a cause. Suggested causes of resurgence for each documented resurgence event were independently evaluated for category and level of evidence by the two reviewers, and disagreements between the reviewers were resolved by consensus.

## **Results**

**Figure 1:**
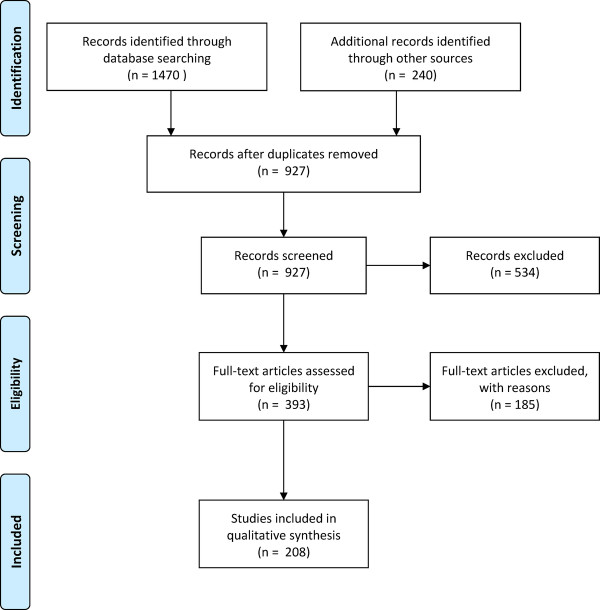
**PRISMA**[78]**systematic review identification, screening, eligibility, and inclusion.**

The database searches returned 1,470 records, and 240 additional records were identified from hand-searching reference lists, producing a total of 927 unique records screened after removal of duplicates (Figure1). Of these, 393 appeared to describe or allude to malaria resurgence and so were assessed for discussion of eligible resurgence events.

Of the 393 reports, 121 (30.8%) were excluded for not mentioning specific examples of malaria resurgence, 39 (9.9%) were excluded for only citing resurgence events by reference to other articles included this review, and 25 (6.4%) were excluded because the events described were determined not to meet the definition of resurgence as described above. For example, increases in malaria were described in regions such as the highlands of north-eastern Tanzania [13] and western New Guinea [14], but these two reports were excluded because evidence was not provided that malaria had previously been actively suppressed from higher levels. Reports of epidemics of malaria that did not appear to represent a sustained trend were also excluded, including outbreaks in Grenada, believed to be sparked by recrudescence of an old infection [15]; Trinidad, where an outbreak of 22 cases occurred in 1994–95 [16] (it was also unclear whether transmission was truly being suppressed before the advent of this outbreak); and Jamaica, where an outbreak occurred 44 years after elimination [17], among others. Thus 208 reports, describing 75 resurgence events in 61 countries, were included in the final analysis (Table1). The events varied greatly in magnitude and duration and occurred from the 1930s through the 2000s. 

**Table 1:** Resurgence events identified by the systematic literature review and their suggested causes

			**Weakening of control activities**					**Technical problems**			**Increasing malaria potential**				
**Place**	**Start**	**End**	**Funding or resource constraints**	**War, disaster, or strife**	**Purposeful cessation**	**Administrative problems, complacency, or poor execution**	**Community non-cooperation**	**Unknown or unspecified**	**Vector resistance**	**Drug resistance**	**Human or mosquito movement**	**Development/ industry changes**	**Socioeconomic weakening**	**Climate/ weather**	**War, disaster, or strife**
**Europe and Middle East**																		
Spain	1936	1943		[47]							[47]	[47]	[47]		[47]			
Italy	1941	1945		[5]											[5]			
Russia	1960	?							[52]		[52]	[52]						
Azerbaijan	1969	1981				[89]												
Afghanistan	1970	1987		[26][60]		[26]			[26]		[26]							
Turkey	1973	1977	[74]			[74]												
Tajikistan	1990	1997		[90][29]														
Azerbaijan	1990	1996	[28][89]	[28][89]							[28][89]		[28][89]					
Turkey	1990	1994									[91]	[91]						
Iran	1991	1999									[92]	[92]	[92]		[92]			
Armenia	1994	1998	[93]	[94]									[94]		[93]			
**Africa**																		
Liberia (Monrovia)	1948	1951	[22]^*^								[22]^*^	[22]^*^						
Kenya (highlands)	1956	1961	[19]^*^			[19]^*^	[19]^*^											
Kenya (Pare-Taveta)	1959	1962			[95]^*^[96]^*^[32]^*^													
Cameroon (Yaounde)	1960	1963			[33]^*^[34]				[34]									
Liberia	1961	?	[22]^*^		[22]^*^													
Zanzibar	1967	1983			[37][97][9]													
Swaziland	1971	1996	[53][21]			[53][21]					[53]^*^[21]	[53]^*^		[21]				
Zambia	1976	2000	[98]					[99]										
Nigeria (Garki)	1974	1975			[35]^*^													
São Tomé and Príncipe	1973	1976		[100]														
Mauritius	1975	1982				[27]					[27]	[27]		[27]				
Madagascar (highlands)	1976	1988	[40]		[40]	[40]												
Kenya (Kisumu)	1977	?	[42]				[42]	[101]										
Ethiopia (Debre Zeit)	1980	1991	[25]^*^		[25]^*^						[25]^*^			[25]^*^				
Mayotte	1981	1984						[102]						[102]				
Sudan (Khartoum)	1981	1993						[51]		[103]	[51][104]			[104]	[104]			
São Tomé and Príncipe	1985	2003	[43]			[100][43]	[43]		[43]									
Zanzibar	1989	1997	[23]^*^			[23]^*^												
Kenya (highlands)	1990	1998								[105][62][63]^*^		[105]		[55]^*^				
Sudan (Gezira)	1990	1994	[5]															
Uganda (highlands)	1990	1994									[106]	[106]						
Zimbabwe	1995	2007	[107]							[108]								
South Africa	1995	2000			[97]				[97]	[109][61]^*^		[109]						
**Asia**																		
China	1960	1970		[31]										[31]				
Sri Lanka	1964	1969	[26]		[20][26]	[26]						[26]						
India	1965	1976	[110][26][18]^*^	[110][18]		[18]^*^[110]	[18]		[111]		[18]	[111,112]						
Pakistan	1967	1972	[26]			[26]			[81]									
Myanmar	1968	2008	[20]			[20]			[20]	[20]	[20]							
Thailand	1970	1981	[20]			[20]	[20]		[20]	[20]	[20]							
Nepal	1971	1986	[20]			[20]		[20]			[20]	[20]						
Bangladesh	1971	1994	[20]	[48]^*^[113]		[48]^*^[20]			[20]	[20]	[48]^*^[113]	[48]^*^	[48]^*^		[48]^*^			
Bhutan	1972	1994			[20]	[20]					[20]							
Vietnam	1979	1991	[49]^*^			[49]^*^				[49]	[49]			[49]				
Pakistan	1980	1992								[64]^*^	[114]^*^	[114]		[58]^*^	[114]^*^			
Sri Lanka	1982	1987				[50]				[50]	[50]	[50]		[50]				
India (Bombay)	1992	1997				[115]						[116]						
Republic of Korea	1993	2000									[46]^*^							
China (Central)	1995	2000	[117]		[118]													
**Americas**																		
Nicaragua	1960	1968	[26]						[26]		[119]							
Paraguay	1961	1967	[120]															
Bolivia	1965	1979	[121][122]			[122]												
Belize	1971	1983	[123]								[45][123]							
Brazil	1974	1992	[123][44]								[124][44]	[44]						
French Guiana	1975	1990				[54]	[41][54]			[54]	[125][41][54]				[41][54]			
Haiti	1976	1982	[123]															
Guatemala	1976	1998		[123]					[45][123]									
Colombia	1976	1998		[126]		[75]												
Dominican Republic	1978	1982	[123]								[123]							
Mexico	1979	1985	[127][128]		[128]	[127]												
Ecuador	1980	1990	[123]		[75]^*^	[127]												
Peru	1981	1998			[75]					[129]	[129]							
Guyana	1983	1991			[75]	[130]					[130]							
Nicaragua	1983	1996	[131]	[26][131]		[131]^*^						[131]			[131]			
Costa Rica	1990	1998	[54]		[54]	[54]					[5][54]	[5][54]						
Belize	1991	1994			[132]^*^[133]													
Suriname	1992	2001		[134]								[134]						
Ecuador	1996	2002	[135]	[135]		[135]								[135]				
Panama	2001	2004				[136]		[137]			[137]							
**Pacific**																		
Indonesia	1963	1973	[26]	[26]					[26]	[26]								
Malaysia (Sabah)	1967	1978				[26]	[26]		[26]	[26]	[26]							
Solomon Islands	1976	1992				[26]		[26]	[26][138]		[26]							
Papua New Guinea	1980	1990						[139]										
Indonesia	1997	2000	[140]^*^[141]^*^			[140]^*^												
Vanuatu	1999	2003	[142]			[142]												

*Indicates in-depth quantitative or qualitative analysis of evidence for suggested causes.

### **Reported causes of resurgence**

Suggested causes of resurgence fell into all three of the general categories. These categories – which were not mutually exclusive – included weakening of the malaria control programme (68/75 = 91%), increases in the intrinsic potential for malaria transmission (44/75 = 59%), and technical problems including drug and insecticide resistance (24/75 = 32%). Subcategories of each are described below.

Only 45 of the 273 (16%) suggested causes for resurgence events identified by the review were classified by reviewers as presenting in-depth qualitative or quantitative analysis to support the assertion. Of these 45 suggested causes, 27 (60%) implicated weakening of malaria programmes, 15 (33%) increases in malaria potential, and 3 (9%) technical problems such as resistance.

### **Weakening of the malaria control programme**

**Figure 2:**
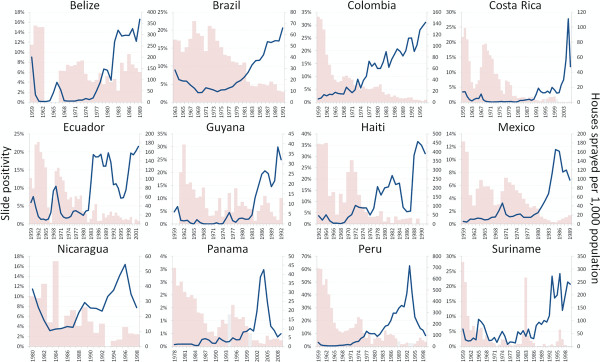
The blue line represents slide positivity (left axis) and the pink bars depict the number of houses sprayed with IRS per 1,000 population [54,79]. Gray bars represent averages of surrounding years where no data on IRS was available in a particular year (otherwise the lack of a bar indicates zero houses sprayed).

Programmatic weakening was attributed to a variety of causes (which are not mutually exclusive), including funding shortages (37/68 = 54%), complacency and other issues with poor execution (32/68 = 47%), war or disaster (17/68 = 25%), purposeful cessation of control activities (17/68 = 25%), community non-cooperation (7/68 = 10%), or unknown or unstated factors (7/68 = 10%). The effects of programmatic weakening are illustrated by the increases in malaria that accompanied the scaling down of indoor residual spraying (IRS) in much of Latin America (Figure2).

**Figure 3:**
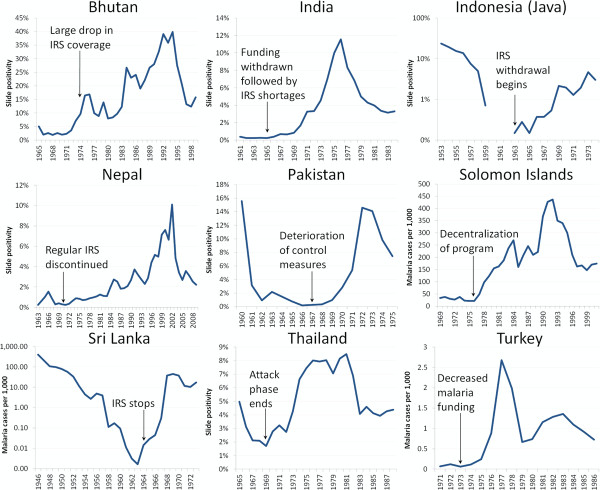
**Malaria resurgence in Asia and Eastern Europe followed weakening – both intentional and accidental – of malaria control programmes.** Resurgence followed general deterioration of control programmes in Bhutan, Indonesia [20,80], Pakistan [81], and Solomon Islands [82]; purposeful weakening of activities in Nepal, Sri Lanka, and Thailand [20,80]; and insufficient funding and resources for vector control in India [20,80] and Turkey [83].

Funding issues were the single most commonly cited reason for resurgence, mentioned in 37/75 (49%) events. Many of these involved time-limited bilateral commitments that funded interventions too costly to continue once the funding period had ended. For example, USAID provided DDT to India for an eradication programme at the end of the 1950s, which led to a very large reduction in the malaria burden, from an estimated 100 million annual cases in the early 20^th^ century to about 100 thousand cases in 1965 [18]. When the USAID commitment ended, however, India proved unable to procure or produce the necessary insecticide to continue the programme, with over a 30% shortfall in 1965–66. Insufficient DDT was likely a key factor resulting in a resurgence of malaria to a peak of 6 million cases by 1976 [18] (Figure3). In the western Kenyan highlands, three WHO-supported sprayings of dieldrin reduced malaria prevalence to 0.5-2.0%, after which the cost of malaria control was transferred to the local government in 1957. Since the cost of spraying was equivalent to the entire health budget for the district, the programme was terminated, and a “striking increase” in malaria had occurred by 1959 [19]. 

Reasons for funding reductions or cessation were not clear for all events, but in several, donors appear to have reallocated funding specifically because successful reductions in malaria burden had occurred. In Thailand, for example, bilateral assistance for malaria control was phased out in 1970 following reductions of the slide positivity rate (SPR) to 1.7% in 1969; within six years the SPR had risen to 8.0% [20]. In Swaziland, the reduction of malaria to the point that it was no longer perceived as a public health problem in the 1950s resulted in significant cut-backs in funding to the WHO-funded control programme, including reductions of the staff from 36 to only seven at the end of the 1960s; significant malaria epidemics involving thousands of cases followed [21].

The reliance of malaria programmes on a few major donors has meant that any change in donor priorities may put continued suppression of malaria at risk. A US-led campaign in Monrovia, Liberia, caused hospital admissions at the public hospital to decrease by about 95% between 1945 and 1947. Thereafter, the programme was deemed too expensive, the budget was cut by 80% in 1948, and by 1950 an assessment concluded that control measures were no longer having any impact on transmission [22]. In Zanzibar, a USAID project in the 1980s was terminated, despite having about $US 1 million in undisbursed funds, due to the perception that the project was a failure [23], and malaria rates on the island of Pemba rose from 23.2% in 1989 [23] to over 60% in 1994 [24]. In Ethiopia, funding from USAID and WHO was halted in 1974 following the overthrow of the government by a military regime [25]. DDT application to households plummeted from a 1974 peak of 117,040 houses to only 8,139 houses in 1985. Incidence increased from 1.1 cases per 1,000 person-years in 1980 to 65.9 cases per 1,000 person-years in 1989 [25]. Similarly, in Indonesia, a DDT programme protecting 17 million people by 1959 was scaled back following withdrawal of assistance from the USA during a tumultuous political period in the early 1960s [26], and malaria increased from <6,000 cases in 1963 to 346,000 in 1973 [20].

In 32/75 events (43%), the weakening of operations for reasons other than funding shortfalls was blamed for subsequent resurgence. In several examples, this weakening was attributed to a sense of complacency within the programme or government resulting from the perception that malaria was no longer a threat. In these examples, commentators do not suggest that insufficient resources were available, nor that programmes were purposefully halted; instead they indicate that the programmes failed to operate sufficiently well despite the apparent availability of resources to do so. In Mauritius, for example, successful certification of elimination was said to have led to a laxness in control: regular testing for malaria was halted and vector control was scaled back, providing an ideal environment for malaria to return following the trigger of a natural disaster [27].

**Figure 4:**
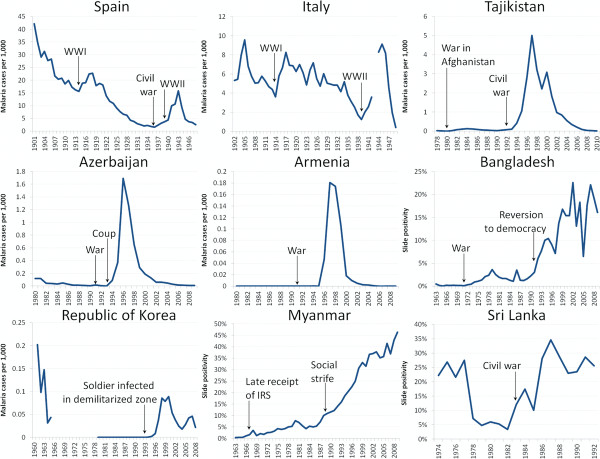
**Malaria resurgence has followed war, population movement, and associated disruptions in Europe and Asia.** Resurgences followed wars and social tumult in Spain, Italy [5], Tajikistan, Azerbaijan, Armenia [83], Bangladesh, Myanmar, and Sri Lanka [20,80], and began in soldiers in the demilitarized zone in Republic of Korea [84,85].

In 17/75 events (41%), war, strife, or natural disaster disrupted programme operations and prevented continued suppression of malaria. For example, in the wake of the dissolution of the Soviet Union, war and strife damaged control efforts throughout the region even as the Soviet support for interventions vanished. The Nagorno-Karabakh civil war in Azerbaijan in the early 1990s interrupted control efforts [28], while war in Tajikistan similarly contributed to disruption and resurgence of malaria [29] (Figure4). In Myanmar, the national malaria control programme lowered incidence from 217 per 1,000 in 1950 to 65 per 1,000 in 1957 (with a prevalence of only 0.11%) [20]. Troubles beset the programme in the 1960s and 1970s, however, and malaria metrics crept upwards; following rebellion and chaos in 1988, slide prevalence doubled from 7.3% in 1987 to 14.0% in 1992, and then continued increasing to 46.4% in 2010 [30]. In China, malaria was reduced from a reported 6.8 million cases in 1954 to 1.58 million in 1959, but following natural disasters, 10 million cases were reported in 1960 [31]. 

**Figure 5:**
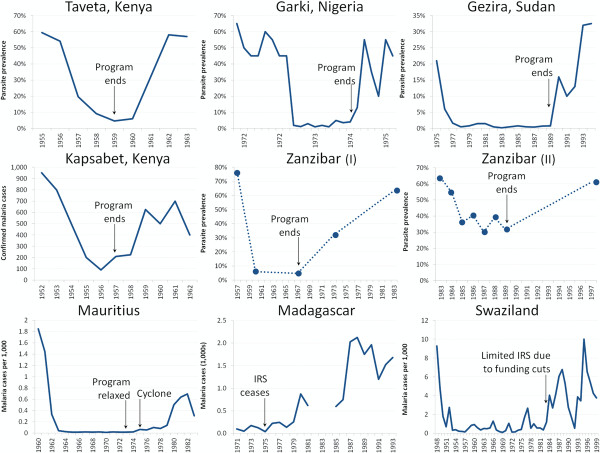
**Malaria resurgence in Africa followed cessation of pilot programmes and relaxation of control activities.** Cessation of pilot programmes in Pare-Taveta [86,87], Garki [35], and Gezira [5] resulted in rapid resurgence to baseline levels, while weakening or halting of control activities in Kapsabet in the western Kenyan highlands [19], Zanzibar (twice) [9], Mauritius [27], Madagascar [40], and Swaziland [88] similarly led to the return of malaria in these areas.

In 17/75 events (23%), malaria interventions were purposefully halted, often because they were intended as time-limited experimental pilots rather than ongoing programmes, especially in sub-Saharan Africa (Figure5). In Central Liberia, for example, WHO and UNICEF started funding one of the first pilot projects intended to examine whether eradication was possible in Africa in 1953; cessation of WHO involvement in 1961 led to rapid deterioration [22]. In Pare and Taveta on the Kenyan-Tanzanian border, malaria was greatly reduced from 1956–59, but it resurged to pre-intervention levels within three years after the pilot ended [32]. In Yaounde, Cameroon, pilot spraying began in 1954 and produced “excellent results” [33], but cessation of spraying in 1960 resulted in complete recovery of the vector [33] with subsequent increases in malaria [34]. And in Garki, Nigeria, prevalence resurged rapidly from <5% prevalence back to a baseline of around 50% following the cessation of the intervention [35]. 

Purposeful decisions to halt successful malaria programmes have also occurred outside of experimental situations. In Zanzibar, a WHO-supported IRS programme reduced malaria prevalence from 76% in 1957 to <5% in 1967 [9], after which Sheik Karume suspended the programme due to his stated belief that Africans were “malaria-proof” [36]. Resurgence rapidly followed cessation of the spraying, and by 1973 prevalence had returned to 54% on the island of Unguja [37]. Elsewhere, countries followed WHO’s eradication guidance and withdrew all vector control measures from regions where malaria had apparently been interrupted [38], in some cases despite lacking sufficiently strong surveillance to maintain elimination in the absence of vector control. In Sri Lanka, for example, a highly successful IRS campaign reduced malaria from 2.8 million cases in 1946 to only 17 (11 of which were imported) in 1963 [20]. Cessation of spraying following the subsequent move from attack to consolidation in 1964 [39], exacerbated by weakened surveillance and increased population movement for mining and agriculture [26], led to a very large increase in malaria incidence, from 150 cases in 1964 to 538,000 in 1969 [20]. In Madagascar, an eradication campaign reduced malaria to very low incidence in a few residual foci by 1960. By 1979, even prophylaxis and treatment centers were closed, and without the checks of either IRS or chemotherapy, the malaria incidence began to rise at a rapid rate [40]. Reintroduction of DDT spraying brought malaria back under control [40].

In 7/75 (9%) of events, resurgence was attributed to decreases in community acceptance or participation in malaria programmes. In French Guiana, it was suggested that resistance of domestic pests, such as cockroaches, to insecticide spraying caused the population to lose confidence in the effectiveness of IRS [41]. Declining compliance with spray campaigns may have contributed to resurgence in Kisumu, Kenya [42], São Tomé and Príncipe [43], and Sabah, Malaysia [26], among others.

### **Increases in malaria potential**

Increases in intrinsic malaria potential were attributed to a variety of causes (which are not mutually exclusive), including movement of humans or mosquitoes (32/44 = 73%), development and land-use changes (19/44 = 43%), climate or weather (11/44 = 25%), war and civil strife (8/44 = 18%), and worsening of socioeconomic conditions (5/44 = 11%).

The most common rationale for why increased transmission potential may have contributed to resurgence involved the movement of humans and mosquitoes and the parasites they carry, cited in 32/75 (43%) events. In Brazil, for example, nearly one million immigrants moved into the Amazon region during the 1970s seeking new farmland and attracted by gold mining in the region. The increase in susceptible individuals in the receptive region, combined with a possible influx of infections from endemic Bolivia, may have contributed to the rapid rise in malaria incidence in the late 1970s and early 1980s [44]. In Thailand at the end of the 1960s, surveillance struggled to detect cases among a mobile population that traveled back and forth from endemic areas [20], while Afghanistan’s nomadic population created similar challenges in the early 1970s [26]. The migration of farmhands from neighbouring endemic countries to Belize in the 1970s fueled transmission [45], while movement of infected mosquitoes across the Demilitarized Zone between the Koreas is believed to have sparked resurgence of malaria in Republic of Korea 14 years after elimination [46]. Movement of refugees and soldiers from wars across countries and decades has been implicated as a cause of malaria resurgence, including in Spain [47], Bangladesh [48], Vietnam [49], Sri Lanka [50], Sudan [51], and Azerbaijan [28].

Nineteen of the 75 events (25%) were at least partially attributed to changes in development or industry, including agricultural development, creation of dams or highways, or other land-use changes. For example, in the USSR, new irrigation and construction of hydroelectric power stations may have increased breeding sites for mosquitoes in the 1960s and 1970s [52]. In Swaziland, development of sugar plantations in the receptive lowveld of the country involved bringing large numbers of potentially infected Mozambican workers into a region where increased agriculture had increased the potential for malaria transmission [53]. Similarly, in Costa Rica, development of the banana industry exacerbated malaria by moving workers from endemic areas into regions with increased suitability for vector breeding [5], while simultaneously reducing the coverage of malaria control programmes to protect them [54].

Eleven of the 75 resurgence events (15%) were attributed at least in part to climate or weather. In Debre Zeit, Ethiopia, malaria rates rebounded in the 1980s after having been successfully suppressed with extensive use of DDT. During this period of increasing malaria rates, positive correlations existed between temperatures and monthly malaria incidence [25]. In Kenya’s western highlands, warming temperatures may have played a role in an increase in epidemic malaria during the 1990s [55]. However, the importance of such factors is controversial given the many other important changes occurring in the region during that time period, including the emergence of chloroquine resistance [56,57]. Increasing temperatures were also suggested as a cause of resurgence in northern Pakistan [58].

Eight of the 75 events (11%) involved increases in transmission potential attributed to war or strife, while another five (7%) were said to be related to a worsening of socioeconomic circumstances. For example, war in Bangladesh in the early 1970s uprooted millions of people and destroyed homes, and, even if the malaria programme had been able to continue its efforts, the lack of stable communities would have made continued control extremely difficult [48]. In 1969, before war began, fewer than 100,000 cases of malaria were reported, but a few years after the war, annual incidence had tripled [20].

### **Technical problems**

Malaria resurgence was attributed primarily to two types of technical problems: vector (14/23 = 61%) and drug (15/23 = 65%) resistance. In 14 of the 75 events (19%), vector resistance to insecticides was suggested as a cause of resurgence. For example, in Nicaragua, an eradication programme failed in 1960 when resistance to dieldrin and DDT was coupled with a shortage of funds, with malaria returning to original levels [26]; DDT was among the cheapest pesticides available, and switching to new insecticides involved an increase in costs at a time when funds were scarce [59]. In Afghanistan, “already deficient operations” were unable to cope with the development of DDT resistance despite an infusion of funding from the USSR in the early 1970s. Malaria incidence there increased from <20,000 cases in 1970 to 127,000 in 1976 [26], and the subsequent invasion of the country by the USSR finished off the malaria control programme [60]. Increasing DDT resistance of mosquitoes in Java may have been an important contributor of resurgence through 1973 [26]. In the USSR, apparent behavioural changes in the vector population were stated to have negatively impacted the programme in the early 1960s [52].

Drug resistance was implicated as a cause of resurgence in 15/75 events (20%). For example, quantitative analysis of factors contributing to resurgence of malaria in South Africa in the 1990s demonstrated that drug resistance was associated with malaria incidence [61], probably because infections that were not effectively treated remained to contribute towards onwards transmission. Similarly, resistance to chloroquine has been suggested as a likely candidate for explaining increases in malaria in the western Kenyan highlands [56,62,63] and northern Pakistan [64].

## **Discussion**

Malaria programmes today face an uncertain future, with the funding available for prevention and treatment projected to decline over the next several years [10]. The results of this systematic review highlight the existential risk to control programmes posed by this deterioration in funding. The review found that the single most common suggested cause of resurgence involved a weakening of malaria programmes following funding disruptions. Leading malaria actors and donors have mobilized to address some of the other resurgence threats identified here, including significant, if still insufficient, efforts to combat the threat of drug [65] and insecticide [66] resistance. However, comparatively limited attention, investment, or action has been devoted to developing practical solutions to financial and operational threats to successful malaria control, despite their apparent importance. At their core, most of these financial and operational hazards result from the same “out of sight, out of mind” paradox: the more successful the programme is, the less visible the disease becomes, and the greater the risk that its funding will be withdrawn or its operations will be conducted lackadaisically [67]. As a result, effective solutions will need to address this root cause, finding ways to sustain the interest of donors, managers, and populations, and increasing the duration and predictability of financial commitments.

This paradox of success is not unique to malaria, and there is considerable experience across public health in continuing vital financing and implementation of programmes in the absence of disease. A primary example is that of vaccination against diseases such as measles, rubella, pertussis, and diphtheria [68]. Similar challenges exist for sustainable immunization campaigns, since parents who no longer perceive the threat of these diseases to their children may choose not to vaccinate [69], while politicians may not see the value of continuing to commit resources for a disappearing disease [70]. The ability of vaccination programmes to achieve continued high coverage rates even in countries where the targeted diseases are no longer visible threats [71] attests to broad understanding of the importance of maintaining these campaigns amongst communities and decision-makers. Success in preventing malaria resurgence requires a paradigm shift from a focus on short-term burden reduction towards an immunization-like programme of routine activities planned and budgeted for the long-term, regardless of the present burden of disease [72].

Nearly all of the 75 resurgence events identified through this review have been ascribed to some aspect of weakening of the malaria control programme, whether because of funding shortages, complacency following successful reductions, or disruptions caused by war or natural disaster. These results suggest that technical problems such as vector resistance appear historically to have been of secondary importance for resurgence to financial and operational factors [73]. The critical causes of resurgence in these events were not the failures of technical solutions; they were the failures of malaria programmes to implement the technical solutions sufficiently well. In India, for example, resistance to DDT, although widely present, was not considered a primary cause of resurgence because of the effectiveness of alternative insecticides and the fact that DDT remained partially effective despite the resistance [18]. In Turkey, despite high levels of resistance, resurgence was attributed to “operational deficiencies stemming from administrative and financial constraints” [74].

These results do not mean that technical problems such as resistance are of no consequence. Observers of malaria resurgence almost always suggest multi-factorial causes. Contributing factors can range from the proximate (e.g., DDT spraying was halted) to the distal (e.g., success against the disease bred complacency and reallocation of funds to more pressing health areas). One of DDT’s chief advantages is its low cost [59], and programmes that could no longer use it due to resistance were required to switch to more expensive insecticides, raising the cost of interventions and making them harder to sustain [75]. If, however, resistance to multiple pesticides was the primary driver of resurgence, it would have been extremely difficult to counteract, since vector control, one of the most effective tools available to malaria control programmes, would have proven useless. Instead, however, regions that made a determined effort were able to continue to make gains against malaria despite the obstacle of resistance. In Indonesia, for example, Gramiccia and Beales blame continued resurgence through 1973 on both insecticide and drug resistance, but note that despite these problems, “intensified anti-malarial measures” were able to greatly reduce malaria following reimplementation post-resurgence [26]. It may also be true that technical problems like resistance develop over longer timelines, and as such could potentially become more important for programmes that successfully establish sustained control measures based on stable funding sources.

This review has a number of limitations. Although systematic, it may have missed any examples of resurgence not documented in the literature or published outside the databases searched. It may also have excluded true resurgence events where insufficient evidence of both an increasing trend and successful prior control was evident in the literature. The magnitudes of resurgence events were not distinguished in this review due to limitations in historical data on malaria incidence and prevalence. The operational importance of a very small increase in malaria burden over time may be quite different from that of a large increase, although both constitute “resurgence” under the definition presented here. It is also plausible that the reports reviewed here may have missed important contributing factors in some cases. The frequency with which certain factors were cited as causes and the paucity of others may be influenced by the research interests of the authors who described them, or perhaps by their affiliations: those associated with national control programmes, for example, may be less likely to implicate programmatic weakening as a cause of resurgence.

This review did not attempt to assess the validity of claims about the causes of resurgence, but instead merely attempted to grade the amount of evidence presented to support each claim. In general, that evidence appears thin: only 16% of claims about the causes of resurgence were found to provide substantial support for their assertions. This review reveals several challenges in evaluating claims about resurgent malaria and its causes, including uneven or unknown malaria baselines, generally poor surveillance during periods of resurgence, and lack of a research infrastructure during the critical periods of interest. Limiting the analyzed causes to only those proposed in articles that provided in-depth analysis of resurgence, however, would not change the results presented here: programmatic weakening was still implicated in the majority (60%) of those articles.

Accordingly, the lesson for today’s malaria programmes is that they must plan carefully to maintain suppressive activities until such a time that no intrinsic potential for transmission remains. It is important to note that many countries have succeeded in doing so. Feachem and colleagues identified 50 programmes that successfully eliminated malaria, predominately during the GMEP [76]. Of these, only four – Armenia, Mauritius, Republic of Korea, and republics of the former USSR – were found in this review to have suffered resurgence in subsequent years. The list of countries that have avoided resurgence include several, such as Taiwan [77], that had high intrinsic transmission potential and were reliant on donor funding to counteract it, belying the notion that such an achievement is beyond the reach of resource-constrained malaria programmes. Contrary to common assumption, the countries currently pursuing malaria elimination and control are not much poorer or weaker than those that have successfully sustained control and elimination in the past. Countries attempting to eliminate today have essentially identical mean GDP per capita to the successful eliminators of the 1960s that have succeeded in avoiding resurgence for decades [76].

Over many decades, socio-economic development and health system strengthening may reduce the intrinsic potential of a region for malaria transmission. In this case, continued control interventions may no longer be necessary to maintain a low burden of malaria. In the interim, however, the global malaria community possesses tools that have been proven to work in reducing illness and death from malaria. Finding ways to maintain the funding, political will, and strong operational capacity to continue to use those tools over the long-term is imperative to ensure that the dramatic progress that has been achieved through international investment is sustained and extended.

## **Competing interests**

The Clinton Health Access Initiative has received funding from the Bill and Melinda Gates Foundation and the Global Health Group at University of California, San Francisco to support national malaria control programmes in maintaining and extending their gains against malaria; GY declares that the Evidence to Policy Initiative has received financial support from the Global Fund to Fight AIDS, Tuberculosis and Malaria.

## **Authors’ contributions**

JMC and OJS conceived of this review. JMC, CC, and AW conducted the review and analysis. DLS, GY, BM, and OJS participated in the interpretation and presentation of the results and contributed to the writing and structure of the manuscript. JMC drafted the manuscript. All authors read and approved the final manuscript.
